# The Dynamic Characteristics of Myocardial Contractility and Extracellular Volume in Type 2 Diabetes Mellitus Mice Investigated by 7.0T Cardiac Magnetic Resonance

**DOI:** 10.3390/jcm11154262

**Published:** 2022-07-22

**Authors:** Chunyan Shi, Hongkai Zhang, Nan Zhang, Dongting Liu, Zhanming Fan, Zhonghua Sun, Jiayi Liu, Lei Xu

**Affiliations:** 1 Department of Radiology, Beijing Anzhen Hospital, Beijing Institute of Heart, Lung & Vascular Diseases, Capital Medical University, Beijing 100029, China; scy0518@126.com (C.S.); zhanghongkai1992@163.com (H.Z.); nzhang_1987@163.com (N.Z.); dongting0530@163.com (D.L.); fanzm120@126.com (Z.F.); leixu2001@hotmail.com (L.X.); 2 Discipline of Medical Radiation Science, Curtin Medical School, Perth 6102, Australia

**Keywords:** magnetic resonance imaging, type 2 diabetes mellitus, myocardial fibrosis, feature tracking, myocardial strain, mice

## Abstract

Type 2 diabetes mellitus (T2DM) is associated with a high prevalence of diastolic dysfunction and congestive heart failure. A potential contributing factor is the accelerated accumulation of diffuse myocardial fibrosis and stiffness. Novel cardiac magnetic resonance (CMR) imaging techniques can identify both myocardial fibrosis and contractility quantitatively. This study aimed to investigate the dynamic characteristics of the myocardial strain and altered extracellular volume (ECV) fraction as determined by 7.0 T CMR in T2DM mice. C57Bl/6J mice were randomly divided into T2DM (fed a high-fat diet) and control (fed a normal diet) groups. They were scanned on 7.0 T MRI every 4 weeks until the end of week 24. The CMR protocol included multi-slice cine imaging to assess left ventricle strain and strain rate, and pre- and post-contrast T1 mapping images to quantify ECV. The ECV in the T2DM mice was significantly higher (*p* < 0.05) than that in the control group since week 12 with significantly impaired myocardial strain (*p* < 0.05). A significant linear correlation was established between myocardial strain and ECV (*p* < 0.001) and left ventricular-ejection fraction and ECV (*p* = 0.003). The results suggested that CMR feature tracking-derived myocardial strain analysis can assess functional abnormalities that may be associated with ECM alterations in diabetic cardiomyopathy, contributing to the study of diabetic therapy effects.

## 1. Introduction

The prevalence of type 2 diabetes mellitus (T2DM) is rising globally [1,2]. Heart failure with preserved ejection fraction (HFpEF) is prevalent in individuals with DM. Accordingly, about 45% of patients with HFpEF have DM, and the prevalence of comorbid DM is increased significantly in those with new-onset HFpEF [3]. Several pathophysiological mechanisms underlying HFpEF were postulated in diabetic patients; however, the development of myocardial fibrosis resulting in the expansion of the extracellular matrix (ECM) is one of the cardinal features [4,5]. Histologically, the myocardium of these patients has been shown to have a high degree of interstitial fibrosis [6,7]. Advanced cardiac magnetic resonance (CMR) imaging techniques have been used to measure the T1 relaxation time of the myocardium [8]. Then, the pre-contrast “native” and post-contrast T1 images of the myocardium were acquired, and myocardial extracellular volume (ECV) fraction was calculated. The ECV calculated by CMR was shown to accurately reflect histologically derived quantification of ECM or interstitial fibrosis [9]. The association of elevated ECV with adverse cardiac outcomes has been reported in studies including diabetic and other patient populations [9,10,11,12].

Recently, the cardiac magnetic resonance-feature tracking (CMR-FT) technique has been proposed as a robust method to evaluate the myocardial strain of the left ventricle (LV) using conventional cine sequence [13,14,15]. It is also speculated that myocardial fibrosis impairs myocardial strain. The early detection of subclinical myocardial dysfunction in patients with diabetes mellitus is essential for recommending therapeutic interventions that can prevent or reverse heart failure and improve the prognosis in these patients. An investigation of the correlation between the myocardial strain and severity of fibrosis may provide new insights into the pathophysiology of patients with T2DM. High-field MR is applied to study the structure and function of the mouse heart [16,17], facilitating the study of cardiac MRI in diabetic animal models in mice. Currently, only a few studies have elucidated the cardiac function images in diabetic mice using high-field MRI. The present study aimed to quantitatively evaluate the LV myocardial deformation and ECV using CMR imaging in mice with T2DM and investigate the association of LV subclinical myocardial dysfunction with myocardium interstitial fibrosis.

## 2. Materials and Methods

### 2.1. Animals

A total of 60 healthy male C57BL/6J mice (weight 13–15 g) (SPF Biotechnology Co., Ltd. Beijing, China) were housed individually at constant temperature and 12/12 h light/dark cycles. Animals received a standard laboratory mice diet and water freely.

### 2.2. Experimental Model and Control Groups

In the control group, mice had free access to normal chow diet (10% calories from fat, Diet D12450J, Research Diets Inc., New Brunswick, NJ, USA), and in the T2DM group to a high-fat diet (60% calories from fat, Diet 12492, Research Diets Inc.). After 4 weeks, the T2DM mice group was induced with a single intraperitoneal injection of streptozotocin (100 mg/kg, Sigma-Aldrich, St. Louis, MO, USA). Streptozotocin was solubilized in a citrate buffer (10 mg/mL; pH = 4.3). The control group mice received only the buffer. After injections, the mice were housed and fed freely with their respective food. After 1 week of injection, blood glucose concentration was determined. Mice with random blood glucose level ≥ 13.9 mmol/L were considered diabetic [18,19] and included in the study. The following study groups were established: non-diabetic control mice (Control; *n* = 30) and mice with type-2 DM (T2DM; *n* = 30). Then, 6 mice were randomly selected from each of the two groups every 4 weeks until the end of week 24. The selected 6 mice were subjected to 7.0T MR scanning to collect the cine MR and T1 mapping images. After scanning, the animals were returned to their original groups for feeding. Before MRI scanning, the mice were treated for approximately 12 h without water deprivation by fasting, weighed, and glucose measured from tail blood. All experimental procedures were approved by our institutional guidelines (No. AEE-2018-020, Beijing, China) and conducted according to the National Institutes of Health (NIH, Bethesda, MD, USA) instructions.

### 2.3. CMR Imaging Protocol

Mice were scanned on a 7.0 Tesla (T) magnetic resonance scanner (Varian, Palo Alto, CA, USA), with four-channel cardiac coils of a mouse. The transit coil used is Rapid 72/99 volume coil, and the receiving coil used is Rapid 4 channel phase array coil. ECG gating and respiratory gating were used when scanning the mice. The scans were performed by dedicated CMR technologists. The mice were anesthetized by inhaling isoflurane gas and fixed in a prone position on an additional wooden board. Then, the ECG device was connected to the limbs and the heart rate and respiratory curve were recorded. Gadolinium-diethylenetriamine penta acetic (Gd-DTPA, Magnevist, 0.5 mM/kg body weight) was injected intraperitoneally during the CMR scan [20]. The body temperature was maintained at 36 ± 0.5 °C, and anesthesia was maintained using 1% isoflurane in O_2_. The blood samples were collected to measure hematocrit (HCT), and body weights were recorded for all animals before starting the diet, and at the beginning of each imaging study.

Cine images—ECG- and respiratory-gated tag-cine sequence was used to acquire cine images in seven to eight slices from base to apex covering the entire LV. Cine-MR images consisted of 15–20 frames per cardiac cycle with 20 images acquired in 11 min. The typical parameters were as follows: field of view (FOV), 25.6 mm × 25.6 mm; acquisition matrix, 128 × 128; time of repetition (TR), 2.4 ms; echo time (TE), 1.4 ms; flip angle, 10°; slice thickness 1 mm with 1 mm interslice gap.

T1 mapping images—All mice received a bolus of intraperitoneal gadolinium-based contrast agent at a dose of 0.5 mmol/kg. An ECG- and respiratory-gated gradient recalled echo (GRE) Look-Locker inversion recovery sequence was utilized to acquire the pre- and post-contrast (~30 min) T1 mapping images at a mid-LV short axis level during the end-diastole [20]. The Look-Locker protocol is based on GEMS sequence, and a linear acquisition is used for k-space. The imaging parameters were as follows: 1 mm slice thickness; FOV, 25.6 × 25.6 mm; data matrix, 128 × 128; TR, 6.5 ms; TE, 3.3 ms; a flip angle, 20°, inversion times, 45; and excitation pulse, 15°. The acquisition time was approximately 8 s/slice to allow full relaxation.

### 2.4. CMR Imaging Analysis

The cine MR and T1 mapping images were analyzed on a dedicated workstation (Vnmr J 4.1, Varian). To determine the LV end-diastolic volume (EDV), the end-diastolic epi- and endocardial LV borders were manually traced on the short axis dataset (after careful exclusion of papillary muscles). The LV-EDV was calculated as the sum of the total slices similar to the end-systolic volume (ESV). The ejection fraction (EF) was derived from the EDV and ESV as follows:

$$\mathrm{Volume}=\overset{\mathrm{all}\;\mathrm{slices}}{\underset{i=1}{\sum }}\left(\mathrm{Endocardial}\;\mathrm{area}\;\times \;\mathrm{layer}\;\mathrm{thickness}\right)$$

EF = (EDV − ESV)/EDV × 100%

A CMR feature-tracking analysis was performed using commercially available software (cvi^42^, Circle Cardiovascular Inc., Calgary, AB, Canada). Digital Imaging and Communications in Medicine (DICOM) images in the short axis of the cine MRI were transferred into the software. Strain analysis was performed to mark the endocardial and epicardial borders of the myocardial slice in each cine MRI image. The trabeculae and papillary muscles were included in the left ventricular cavity. The two-dimensional (2D) left ventricular global peak radial strain (LV-GRS) and circumferential strain (LV-GCS), left ventricular peak systolic global radial strain rate (LV-S-GRSR) and circumferential strain rate (LV-S-GCSR), and left ventricular peak diastolic global radial strain rate (LV-D-GRSR), and circumferential strain rate (LV-D-GCSR) were calculated based on the short-axis cine MRI images.

The ECV was calculated using T1 maps. The T1 time for the blood pool was measured after careful exclusion of the papillary muscles. The ECV fraction was calculated using the following formula:

ECV = λ (100 − hematocrit)

Λ = (1/Postcontrast Myocardial T1 − 1/Precontrast Myocardial T1)/ (1/Postcontrast Blood Pool T1 − 1/Precontrast Blood Pool T1)

HCT was measured from the blood samples obtained from the mice before scanning.

### 2.5. Statistical Analysis

Data were analyzed using GraphPad Prism software (version 8.0, GraphPad Software, San Diego, CA, USA). The normality and homogeneity of the measurement data were tested using the Shapiro–Wilk and *F* tests, respectively. Continuous values were presented as means ± standard deviation. The differences in heart rate, respiration, body weight, blood glucose, EDV, ESV, and EF between the T2DM and control groups were analyzed using an unpaired *t*-test. The differences in strain and ECV values between the two groups at each time point were also analyzed using an unpaired *t*-test. The correlation between ECV and strain value was assessed using Pearson’s correlation coefficients. A *p*-value < 0.05 was considered statistically significant.

## 3. Results

### 3.1. Animal Characteristics and the Results of MRI Parameters

Table 1 shows the animal characteristics and the results of the MRI imaging parameters.

Body weight was significantly higher in the T2DM mice at each time point post-diet and increased in a time-dependent manner (*p* < 0.05 vs. age-matched controls). We also noted a slight increase in body weight in the control group, probably due to normal growth at their age. The T2DM mice were hyperglycemic during post-diet as measured from the fasting blood glucose (*p* < 0.05 vs. age-matched control). No significant differences were detected in the heart rate and respiration between the two groups of mice during the study (Table 1).

Left ventricular end-systolic volume (LV-ESV) was significantly increased in the T2DM mice during 16–24 weeks post-diet (*p* < 0.05 vs. age-matched control). The mean left ventricular ejection fraction (LVEF) at the 24 weeks post-diet was 52 ± 5% in the T2DM mice and 62 ± 6% in the control mice; the difference was statistically significant. No statistically significant differences were detected in the left ventricular end-diastolic volume (LV-EDV) measurements between the two groups of mice (Table 1).

T1 values of left ventricular myocardium and blood pool post-contrast were significantly decreased in the T2DM mice during 16–24 weeks post-diet (*p* < 0.05 vs. age-matched control) (Table 1).

### 3.2. Comparison of the Myocardial Strain Parameters between the T2DM and Control Mice

**Figure 1:**
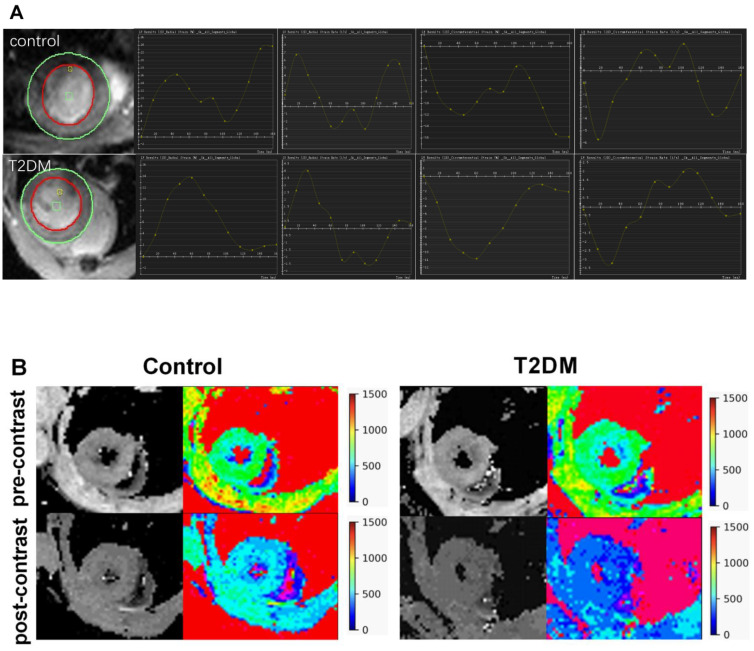
Feature tracking strain analysis and ECV map in representative T2DM mice and control. (**A**): The first column on the left shows the cine image of the mouse heart. The curves show the change in the left ventricular myocardial strain and strain rate with the cardiac cycle. (**B**): T1 mapping images and pseudocolor maps of mouse hearts. ECV: extracellular volume.

Figure 1 illustrates the strain analysis and ECV map in representative T2DM and control mice. The average ECV value was substantially evaluated in the papillary slice.

**Figure 2:**
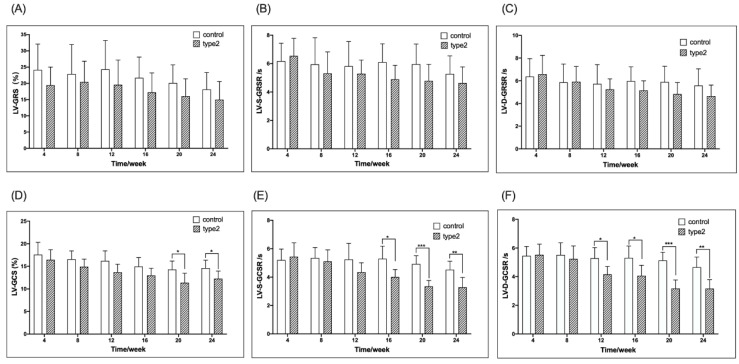
Timely/weekly comparison of the LV-GRS, LV-S-GRSR, LV-D-GRSR and LV-GCS, LV-S-GCSR, LV-D-GCSR between the T2DM mice and controls (**A**–**F**). LV-GRS, left ventricular global radial strain rate; LV-S-GRSR, left ventricular systolic global radial strain rate; LV-D-GRSR, left ventricular diastolic global radial strain rate. LV-GCS, left ventricular global circumferential strain rate; LV-S-GCSR, left ventricular systolic global circumferential strain rate; LV-D-GCSR, left ventricular diastolic global circumferential strain rate. T2DM: type 2 diabetes, * *p* < 0.05, ** *p* < 0.01, *** *p* < 0.001.

Figure 2 shows the weekly comparison of the LV-GRS, LV-S-GRSR, LV-D-GRSR, LV-GCS, LV-S-GCSR, LV-D-GCSR, and ECV between the T2DM and control mice. Compared to the control mice, the T2DM mice had a significantly lower LV-GCS, LV-S-GCSR, and LV-D-GCSR at 20, 16, and 12 weeks, respectively (LV-GCS, −11.38 ± 2.08 vs. −14.30 ± 1.89, *p* < 0.05; LV-S-GCSR, −4.01 ± 0.52 vs. −5.29 ± 0.89, *p* < 0.05; LV-D-GCSR, 4.16 ± 0.55 vs. 5.29 ± 0.74, *p* < 0.05, respectively) (Figure 2D–F). No significant difference was detected in LV-GRS, LV-S-GRSR, and LV-D-GRSR between the T2DM and control mice at all the time points (Figure 2A–C).

### 3.3. Comparison of EF and ECV between the T2DM Mice and Controls

**Figure 3:**
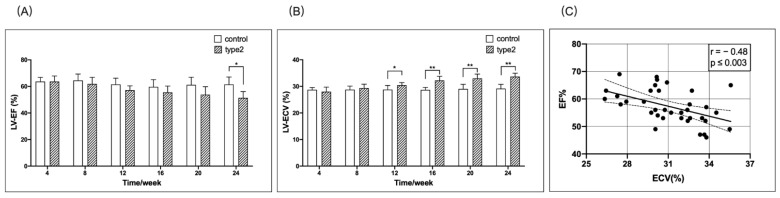
Timely/weekly comparison of the LV-EF and LV-ECV between the T2DM mice and controls, and the correlation between the EF and ECV. The mean LV-EF in the T2DM mice was decreased starting at 8 weeks, but reached significant difference (* *p* < 0.05) at 24 weeks when compared to the matched control mice (**A**). The mean LV-ECV was significantly higher (** *p* < 0.01) in the T2DM mice than that in the matched control mice from 12–24 weeks (**B**). There was a significant correlation between LV-EF and LV-ECV (**C**). EF, ejection fraction; ECV, extracellular volume.

The mean LV-EF was decreased significantly in the T2DM mice at 24 weeks post-diet than in the matched control mice (Figure 3A). Additionally, the mean LV-ECV was increased significantly in the T2DM mice compared with the matched control mice from 12–24 weeks (Figure 3B). Furthermore, we observed a moderate correlation between LV-EF and the LV-ECV (Figure 3C).

### 3.4. Correlation between ECV and LV-GRS, LV-S-GRSR, LV-D-GRSR, LV-GCS, LV-S-GCSR, and LV-D-GCSR

**Figure 4:**
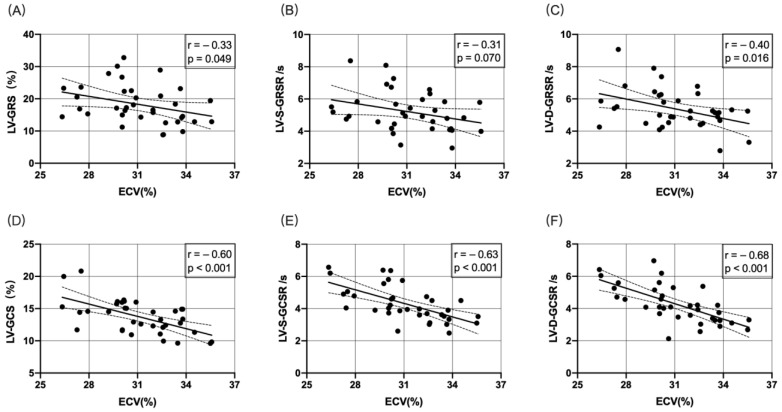
Correlation between ECV and LV-GRS, LV-S-GRSR, LV-D-GRSR, LV-GCS, LV-S-GCSR, and LV-D-GCSR (**A**–**F**). A significant negative correlation was observed between ECV and LV-derived myocardial strain analysis (*p* < 0.05).

Figure 4A–C shows the correlation between ECV and LV-GRS, LV-S-GRSR, or LV-D-GRSR. A significant negative correlation was established between ECV and LV-GRS, LV-S-GRSR, or LV-D-GRSR (*r* = −0.33 to −0.40; *p* < 0.05). Figure 4D–F demonstrates the correlation between ECV and LV-GCS, LV-S-GCSR, or LV-D-GCSR. A significant negative correlation was established between ECV and LV-GCS, LV-S-GCSR, and LV-D-GCSR (*r* = −0.60 to −0.68; *p* < 0.05).

## 4. Discussion

A proposed mechanism for diabetic cardiomyopathy is the deposition of collagen in the ECM due to increased expression of tumor necrosis factor-beta (TGF-β), and connective tissue growth factor and decreased expression of matrix metalloproteinases [21,22,23]. Histological and CMR-based studies have shown an association of DM with ECM expansion [6,7,10,24]. ECV was used as a non-invasive imaging biomarker for ECM expansion. Next, we evaluated the significance of the association of myocardial strain with elevated ECV in T2DM mice within 24 weeks. The current study confirmed the correlation between decreased contractility and increased ECV. Thus, we speculated that the convenient analysis of myocardial contractility underlies the deposition of myocardial fibrosis, which plays a significant role in predicting diabetic cardiomyopathy.

ECV may represent a novel non-invasive biomarker to evaluate the severity of diabetic heart disease. With advancing diabetes, collagen deposition increases in the ECM [25]. The effect of increased ECV on the myocardium is not well-understood. Rommel et al. [26] evaluated the association between elevated ECV and LV stiffness in patients with HFpEF, and depicted that diffuse myocardial fibrosis independently predicts invasively measured LV stiffness in HFpEF. In this study, elevated ECV was associated with decreased LV contractility. However, T1 and calculated ECV via CMR are non-specific markers that may not only reflect fibrosis, but also edema and inflammation at the early stage of diabetes. The increased T1 values of left ventricular myocardium and blood pool post-contrast in the T2DM mice suggest that the post-contrast T1 changes may be a complementary assessment to feature tracking strain analysis. Additionally, the left ventricular global circumferential strain and strain rate (LV-GCS, LV-S-GCSR, and LV-D-GCSR) were significantly decreased compared to the control group throughout the observation period, whereas the changes were not significant in the radial strain and strain rate (Figure 2). This phenomenon illustrated that the circumferential strain and strain rate were superior to the radial strain and strain rate while characterizing the cardiac contractility in diabetic cardiomyopathy. Moreover, among the three circumferential strain values, the strain rate was statistically different at an earlier time point than strain compared to the controls, wherein the peak diastolic strain rate occurred earlier than peak systolic strain rate, consistent with the time point (12 weeks) when the difference was detected in ECV. Thus, LV-D-GCSR may be a useful indicator for depicting LV contractility in diabetes.

In the present study, the circumferential strain and strain rate of the left ventricle in the T2DM group were significantly reduced compared to those in the control group with prolonged observation time, indicating that a continuous state of hyperglycemia reduces myocardial circumferential systolic function. Our previous findings showed that the degree of diffuse fibrosis of circular muscle aggravated with the progression of diabetes, which was consistent with the decreasing change in circumferential strain. Combined with the fact that circumferential strain was determined by mid myocardial circular muscle in a previous study [27], we speculated that the mid myocardial circular muscle was sensitive to the hyperglycemic state. Radial strain is in the 30–40% range during systole and reflects wall thickening towards the center of the LV [28]. In this study, the radial strain and strain rate of the left ventricle of mice in the T2DM group did not show significant differences compared to the control group during the observation period. These findings could be attributed to the following reasons: first, the contraction of the sarcomeres occurs along the myofibers, hence active contraction is only longitudinal and circumferential, whereas radial thickening is not a primary phenomenon, but a consequence of fiber rearrangement [29]; second, the thickness of the LV wall in the mouse is only 1.5–1.8 mm [30], making peak radial strain measurements potentially inaccurate. Therefore, peak radial strain may not accurately respond to alterations in myocardial mechanics in diabetic mice.

Compared to the control group, elevated ECV and decreased LV contractility were observed simultaneously (week 12) in this study. Next, the correlation between myocardial strain and rate, EF value, and ECV was analyzed. The results showed that the strain, strain rate, and EF have a negative correlation with ECV (*p* < 0.0001) (Figure 3 and Figure 4). The correlation between LV-D-GCSR and ECV was highly significant (r = 0.68, *p* < 0.001). Therefore, we concluded that LV-D-GCSR is a promising indicator to assess the association of cardiac dysfunction with myocardial fibrosis. This finding needs to be validated in future studies with a large sample size.

Previous studies confirmed that the myocardial strain is superior to EF values for the evaluation of cardiac dysfunction, especially for HFpEF [31,32]. The results of this study exhibited that the GCS and GCSR of the left ventricle in diabetic mice differed significantly from the value of LVEF (20 weeks vs. 24 weeks), which further confirmed the above conclusion. In summary, CMR-FT technique can quantitatively evaluate cardiac deformation in diabetes at an early stage.

Furthermore, compared to ECV, the acquisition of myocardial strain is simple, clinically feasible, and based on cine sequence, without additional scanning sequence and contrast agent injection. The present study confirmed a strong negative correlation between myocardial strain and ECV, revealing the intrinsic link between myocardial strain and myocardial fibrosis. This provided a simple tool to assess the progression of myocardial fibrosis in diabetic cardiomyopathy and monitor the anti-fibrotic therapy. Thus, these imaging markers seem promising for the detection of pre-clinical cardiomyopathy in patients with T2DM. Therefore, the current findings could lay the foundation for future research on patient data.

Nevertheless, the present study has some limitations. First, some studies have shown that in patients with T2DM with preserved ejection fraction and no complications, the longitudinal strain of LV is impaired; however, its circumferential and radial strain is preserved. This study was restricted by scanning range, and no longitudinal strain was measured. Thus, results need to be interpreted with caution. Second, the sample size was small, and the myocardial strain and other changes were analyzed over 24 weeks. Thus, a large cohort is required to validate the observed outcomes. Third, at each timepoint, six mice were randomly chosen, but not the same mice selected each time, so we ignored the individual differences of mice, although the results of the self-control design may be more convincing. Fourth, strain and mapping data might be influenced by the very small myocardial thickness in mice and very high heart rates. Lastly, the mice model over 24 weeks only covers the early stage of the development of a diabetic cardiomyopathy.

## 5. Conclusions

Taken together, in T2DM mice, a good correlation was established between myocardial strain and ECV, suggesting that the CMR-FT-derived myocardial strain might be a useful and innovative non-invasive imaging biomarker to evaluate the ECM alterations in diabetic cardiomyopathy. The early recognition of ECM expansion via a simple CMR-FT technique in diabetic patients may lower the mortality associated with elevated ECV by initiating appropriate anti-fibrotic therapy. In addition, this animal model depicts early changes of the myocardial tissue character with ECV and functional parameters in the early development of a potential diabetic cardiomyopathy may be monitored.

## Figures and Tables

**Table 1:** Animal characteristics of both study and control group mice during the 24-week study.

	4 Weeks		8 Weeks		12 Weeks		16 Weeks		20 Weeks		24 Weeks	
	Control (*n* = 6)	T2DM (*n* = 6)	Control	T2DM	Control	T2DM	Control	T2DM	Control	T2DM	Control	T2DM
Heart rate, bpm	394 ± 36	438 ± 47	391 ± 33.55	384 ± 43	416 ± 29	407 ± 47	401 ± 27	371 ± 38	404 ± 45	389 ± 31	395 ± 41	384 ± 38
Respiration, bpm	37 ± 4	37 ± 6	38 ± 4	36 ± 5	38 ± 2	36 ± 4	37 ± 3	35 ± 4	36 ± 4	37 ± 4	35 ± 3	39 ± 6
Bodyweight, g	23.9 ± 0.5	26.7 ± 0.8 *	25.7 ± 0.6	28.8 ± 1.3 *	26.9 ± 0.7	32.7 ± 1.2 *	29.1 ± 0.7	37.2 ± 0.8 *	28.2 ± 0.6	36.6 ± 0.6 *	27.8 ± 0.5	35.7 ± 0.8 *
Blood glucose, mmol/L	5.9 ± 0.9	17.6 ± 1.0 *	6.1 ± 1.1	18.3 ± 1.0 *	6.4 ± 0.7	19.4 ± 0.7 *	5.8 ± 0.7	17.8 ± 1.0 *	6.5 ± 0.9	17.7 ± 1.3 *	6.1 ± 1.1	17.4 ± 1.2 *
LV-EDV, μL	46.72 ± 6.66	50.48 ± 7.16	57.13 ± 11.52	47.38 ± 6.16	52.19 ± 13.24	56.2 ± 10.31	50.59 ± 9.87	59.66 ± 7.10	59.55 ± 9.55	66.41 ± 5.55	58.67 ± 4.34	59.37 ± 6.88
LV-ESV, μL	16.9 ± 2.09	18.2 ± 2.15	20.78 ± 6.61	18.02 ± 2.32	20.18 ± 5.95	24.37 ± 5.69	20.2 ± 3.12	26.3 ± 2.31 *	23.28 ± 4.92	30.86 ± 6.09 *	22.48 ± 3.37	28.88 ± 4.96 *
LV-EF	0.64 ± 0.03	0.64 ± 0.04	0.64 ± 0.05	0.62 ± 0.05	0.61 ± 0.05	0.57 ± 0.03	0.60 ± 0.05	0.56 ± 0.05	0.61 ± 0.06	0.54 ± 0.06	0.62 ± 0.06	0.52 ± 0.05 *
T1 Myocardium pre-c. (msec)	1007 ± 23	983 ± 34	1021 ± 31	989 ± 33	1000 ± 21	999 ± 24	1013 ± 25	1020 ± 23	997 ± 18	1038 ± 23	1009 ± 15	1047 ± 26
T1 Blood pre-c. (msec)	1177 ± 69	1112 ± 54	1229 ± 37	1118 ± 44	1193 ± 72	1107 ± 27	1187 ± 38	1140 ± 24	1162 ± 47	1194 ± 28	1231 ± 36	1162 ± 26
T1 Myocardium post-c. (msec)	648 ± 26	661 ± 15	641 ± 17	640 ± 21	631 ± 24	621 ± 27	647 ± 30	575 ± 24 *	666 ± 18	560 ± 308 *	634 ± 38	550 ± 17 *
T1 Blood post-c. (msec)	543 ± 25	556 ± 21	546 ± 13	540 ± 12	534 ± 31	517 ± 32	548 ± 32	477 ± 33 *	559 ± 14	465 ± 30 *	541 ± 31	461 ± 16 *
Hct (%)	49.1 ± 2.0	48.7 ± 1.7	49.7 ± 1.1	49.0 ± 1.7	49.3 ± 1.4	48.0 ± 1.8	49.4 ± 1.6	47.5 ± 1.5	46.3 ± 1.1	47.3 ± 1.6	48.3 ± 2.3	48.4 ± 1.3

Data are presented as the mean ± standard deviation. bpm, beats per minute; LV-EDV, left ventricular end-diastolic volume; LV-ESV, left ventricular end-systolic volume; LV-EF, left ventricular ejection fraction; pre-c., pre-contrast; post-c., post-contrast; Hct, hematocrit. * *p* < 0.05 vs. Control at the same time point.

## Data Availability

All the data and analyses are available upon request.
